# Pattern of Otorhinolaryngological Admissions via Emergency Unit in a Suburban Tertiary Center

**Published:** 2015-09

**Authors:** Taiwo O. Adedeji, Olusola A. Sogebi, James E. Tobih

**Affiliations:** 1 Department of Otorhinolaryngology Head and Neck Surgery, LAUTECH Teaching Hospital, Osogbo, Osun state, Nigeria;; 2 ENT Unit, Department of Surgery, College of Health Sciences, Olabisi Onabanjo University, Sagamu, Nigeria

**Keywords:** ENT emergency admissions, upper airway obstruction, pharyngo-esophageal FBs, epistaxis, corrosive ingestions, esophageal denture impaction

## Abstract

**Background:**

Patients with ORL lesions sometimes present to the general emergency room. This study reviews the common ENT admissions via emergency room in a sub-urban town in Nigeria

**Methods:**

A retrospective study spanning five years from January 2009 to December 2013

**Results:**

A total of 211 cases consisting of 131 (62.1%) males with male: female ratio 1.6: 1 and a mean age of 32.8 ± 22.4 years. About a quarter of the patients were children, the peak age was 21-40 years (in 37%). The common indications for emergency otorhinolaryngological admissions were Epistaxis (16.1%), Nasal/facial trauma (14.7%), pharyngo-esophageal foreign bodies (13.3%) and upper airway obstruction (8.1%). Majority 16 (57.1%) of the Pharyngo-esophageal FBs occurred in children. Most of the airway obstructions in children were due to juvenile recurrent respiratory papillomatosis while laryngeal cancer was the major cause among the adult. Sixty percent had surgical procedures, 86.7% had satisfactory outcome and mortalities were recorded in 1.4%.

**Conclusion:**

Majority of causes for ORL admissions via emergency unit are of pharyngo-esophageal origin. There is apparent reversal of the otological origin trend in ENT admissions via A&E unit.

## INTRODUCTION

Emergency room services are important and integral part of medical practice (1). Efficiency of such services can be used as an indirect indicator of the quality of health care services provided (2-4). Despite proliferation of sub-specialties and disciplines within the medical practice, most hospitals have a common/general emergency room, manned by medical-officers that initially sort, evaluate and initiate management of patients before the specialists are invited. Patients with ORL lesions sometimes present to the general emergency room. Studies have shown that the workload of otorhinolaryngologist (ENT specialists) have been on an increase due to increasing number of cases of ENT emergencies (2, 5, 6). This is due to increasing incidence of road traffic accidents and industrial disasters that affect the facial, orofacial and cervical regions of the body, creating a challenge to the attending ENT surgeon (2). Immigration and increase in life-expectancy are other factors suspected to have probably influenced this increase (7). ORL emergencies can be particularly devastating and may carry grave consequences.

The ear and nose harbor some delicate and important special sensory organs whose injuries can seriously affect functioning of the individual. Furthermore, their proximity to the eyes and brain make early diagnosis and management of lesions in this region imperative. Also the nose and throat form part of the upper airway whose obstruction could portend a threat to life. A study from Ghana showed that the most common causes of mortality among ENT patients were respiratory tract obstruction, intracranial complications of chronic suppurative otitis media and foreign body in the upper aerodigestve tract (1).

The challenge of effective management will substantially require the services of the appropriate personnel, who can decipher the seriousness or urgency of the patient’s complaints, and also decide on patients who will subsequently require admission.

While a sizeable number of these ORL patients will require hospital admission after initial treatment, it is not all patients that present in the emergency room who will require emergency care or subsequent ward admission. Timsit *et al* (8) reported that despite a rise in the number of ENT emergencies, only 10% of the consultation in the ENT emergency unit required admission. In USA, it was reported that most of the consultations at the ENT emergencies were for trivial lesions that required treatment but not admission (9).

This study therefore assesses the pattern and distributions of ear, nose, and throat admissions via the emergency department as seen in a tertiary health facility in a sub-urban town in Nigeria. Knowledge of these patterns will assist in proper deployment of personnel, direct our focus on the area of training and management thus strengthening our health systems.

## METHODS

The study was a retrospective, descriptive study. The subjects studied were patients admitted into the ENT ward via the general A&E unit at Ladoke Akintola University of Technology Teaching Hospital, Osogbo, Osun State, Nigeria.

The study was for five-year period between January 2009 and December 2013. Clinical records of the patients from accident and Emergency unit and from the ENT ward were obtained. Excluded from the study were patients whose case records could not be located, those with incomplete information, patients whose condition did not require admission and referrals from other departments.

The clinical information retrieved from patients’ hospital records included socio-demographic records like age, sex and occupation, clinical diagnosis, management and outcome.

The information obtained was fed into a spreadsheet and the data generated was analyzed using SPSS version 14 (Illinois, USA). Descriptive analysis was done as proportions and the results presented in tabular forms and charts.

## RESULTS

**Figure 1:**
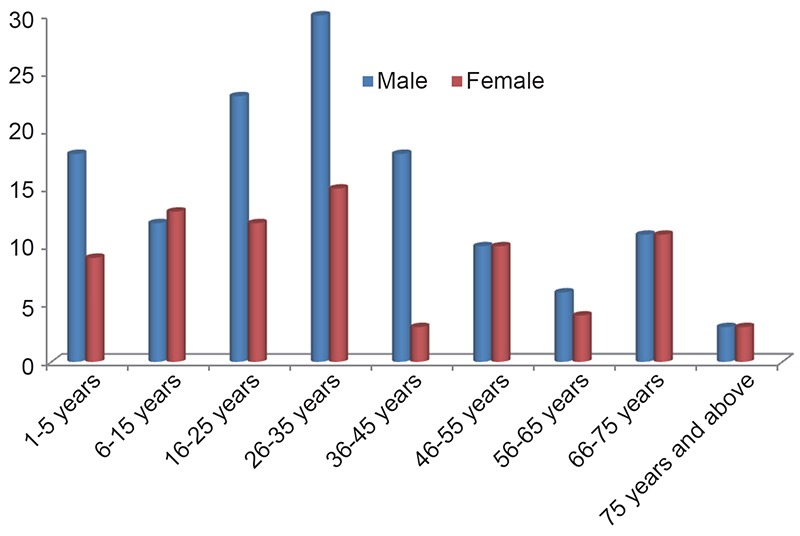
Age and sex distribution of the patients.

There were 764 cases of ENT conditions that presented via the A & E unit, 211 (26.6%) of these required ward admission for otolaryngological management. There were 131 (62.1%) males with male: female ratio 1.6: 1.The age ranged from 1 to 90 years with a mean age of 32.8 ± 22.4 years. About a quarter (24.6 %) of the patients were children aged 15 years and below. Figure 1 shows the age and sex distribution of the patients. Considering lesions in relation to ENT regions pharyngo-esophageal lesions constituted the highest proportion (43.2%) of all cases of admission. The common indications for otorhinolaryngological admissions were Epistaxis (16.1%), Nasal/facial trauma (14.7%), pharyngo-esophageal foreign bodies (13.3%), and upper airway obstruction (8.1%) as shown in Table 1. The indications for ORL admissions according to category of patient (children or adult) is shown in Table 2. Among the children, the leading indications for admission were pharyngo-esophageal FBs impaction (in 30.8%) and upper airway obstructions (in 17.3%). The common indications for admission in the adults (>15 years) were epistaxis (20.8%) and nasal/facial trauma (16.4%). One hundred and twenty six patients (59.7%) had various surgical procedures. One hundred and eighty three (86.7%) had satisfactory outcome following treatment while there were 3 (1.4%) mortalities. The other complications are shown in Table 3.

**Table 1:** Indications for ORL admission via emergency room in all patients

Diagnosis	Number	Percentage

**Ear**		
Vertigo	13	6.2
Basal skull fracture	10	4.7
Otitis externa	6	2.8
Acute mastoiditis/mastoid abscess	3	1.4
Pinna laceration	3	1.4
**Total**	**35**	**16.5**
**Nose**		
Epistaxis	34	16.1
Nasal/facial injuries	31	14.7
Septal hematoma	1	0.5
**Total**	**66**	**31.3**
**Throat/ Pharyngo-esophagus**		
Pharyngo-esophageal FBs	28	13.3
Upper airway obstruction	17	8.1
Corrosive ingestions	14	6.6
Denture impaction	13	6.2
Acute tonsillitis	10	4.7
Acute pharyngitis	5	2.4
Peritonsillar abscess	4	1.9
**Total**	**91**	**43.2**
**Neck**		
Neck space infections	7	3.3
Neck trauma / cut throat	3	1.4
**Total**	**10**	4.7
**Others**	9	**4.3**
**Total**	211	100.0

**Table 2:** Major indications for admissions via A & E according to category of patient

Children (≤15 years)	(N=52)	Adult (≥15 years)	(N=159)

Pharyngo-esophageal FBs	16 (30.8%)	Epistaxis	33 (20.8%)
Upper airway obstruction	9 (17.3%)	Nasal/ facial trauma	26 (16.4%)
Corrosive ingestion	7 (13.5%)	Pharyngo-esophageal FBs	25 (15.7%)
Nasal/facial trauma	5 (9.6%)	Vertigo	13 (8.2%)
Acute tonsillitis	3 (5.7%)	Skull base fracture	10 (6.3%)
Otitis externa	3 (5.7%)	Upper airway obstruction	8 (5.0%)
Mastoiditis/mastoid	2 (3.8%)	Corrosive ingestion	7 (4.4%)
abscess	7 (13.5%)	Acute tonsillitis	7 (4.4%)
		Neck space infection	6 (3.8%)
		Acute pharyngitis	4 (2.5%)
		Others	20 (12.6%)
Total/p>	52 (100%)		159 (100%)

**Table 3:** Treatment modalities & complications observed among ORL admissions via A & E

Treatment modalities	Frequencies (%) (N = 211)	Complications (N = 27)	Frequencies (%)

Observation	17 (8.1)	Hearing loss	8 (29.6)
Medical management	67 (32.2)	Facial palsy	6 (22.2)
Nasal packing	29 (13.7)	Esophageal stricture	5 (18.5)
FBs removal under GA	41 (19.4)	Death	3 (11.1)
Tracheostomy	16 (7.6)	Hoarseness	2 (7.4)
Suturing	23 (10.9)	Tracheostomy dependence	2 (7.4)
I & D	16 (7.6)	Nasal deformity	1 (3.7)
Cervical collar	1 (0.5)		
Total	211 (100.0)		27 (100)

I & D, Incision and drainage; FB, Foreign bodies; GA, General anesthesia.

## DISCUSSION

Otorhinolaryngological lesions are frequent presentations in accident and emergency units of hospitals in many communities (1-3). The proportions of these patients that may require subsequent admission may however be small (8, 9). In the present study, approximately one in every four patients (26.6%) that presented in the emergency room for ORL lesions actually required an admission for ORL management. This is marginally higher than the reported range of between 10% and 25% of the cases of ORL emergencies that actually qualified for admission for in-patient management (7-11). This may be related to the fact that many of the patients with minor ORL lesions like ear discharge and foreign bodies in the ear and nose would be treated on outpatient bases. Yojana *et al* (2) however reported 75.7% admission rate for their patients that presented in the ENT emergency, 84.0% of whom had facio-maxillary injuries. The foregoing underscores the fact that type and severity of lesions should be the yardstick for admission for in-patient management, rather than mere presentation. An initial evaluation of patients by ENT specialists at the general A&E units should be emphasized in order to avoid needless admissions. by general duty medical officers. In a study relating admissions and duration of stay in an ear, nose and throat department, Fernández *et al* reported that up to 17.8% of non-elective ENT admissions were inappropriate (12).

The male preponderance seen in the study is similar to the findings from previous published studies (1, 2, 13). Male preponderance may be activity related especially among the children while among the adults in the developing countries, the fact that men are usually the breadwinners, and are more prone to traumatic experiences may be a contributing factor. Similar studies in developed countries however did not portray gender inequality possibly due to their liberality with equal job opportunities and exposure for both genders (2, 6, 7). The mean age of patients of 32.8 years noted in our study was similar to a mean age of 32 years reported in India (4) but higher than 25.5 years reported in Ghana (1). While some (2) including our study reported the peak incidences at the ages of between third and fourth decade of life, some other studies reported peak incidence at the age in the first decade of life (1, 13). The factor responsible may be due to the pattern of presentations. Most pediatric cases that were diagnosed as otitis media and foreign bodies’ impactions in the ear and nose were not included in our study as they did not require admission.

The trend that most (43.2%) of ENT admission via emergency room was due to pharyno-laryngeal lesions and the least (16.5%) due to otologic lesions seen in this study is a reverse of predominant otologic lesions reported by some other authors (7, 14, 15). This might have been due to our exclusion of common otologic complaints of FB insertion in the ears from our study (7, 14, 15).

Although similarities occur between our findings and that of the study done in Ghana regarding major causes of otorhinolaryngological admissions, there was, a higher proportion of naso-facial injuries in our study. This may not be unconnected with our practice of managing facial, maxillary and mandibular injuries as a stop-gap for the absence of maxillo- facial or dental unit in our center. Another factor may be due to the increasing incidence of road traffic accidents consequence of bad roads or carelessness of the drivers. There is need for better road use awareness amongst the populace while governmental effort on road maintenance and safety measures should be stepped up. A researcher had even corroborated the increasing prevalence of facial, head and neck injuries among adults in Nigeria (15). All patients with cases of nasal fractures were reduced as per using standard protocol, those with simple maxillary or mandibular fractures had inter-maxillary fixation with good outcome while those with complex zygomatic fractures were referred to maxillofacial surgeon.

Our findings among the children revealed that pharyngo-esophageal FBs was the major indicator for ENT admission via emergency unit, similar to previous reports (16-23). Children are inquisitive and explorative in nature, and have a tendency to insert FBs into the craniofacial orifices with predisposition to upper airway obstruction (16). It was noted that none of the FBs impacted in the aero digestive tract was a coin, an apparent reversal of trends noted in previous studies (1, 17). Coins are no longer accepted universally as a means of exchange in Nigeria during the period of this study, in contrast to what obtained few decades ago and that may account for our present findings. The major FBs found in these children were metals, bones and plastic/toys.

Foreign bodies in the aero digestive system are preventable causes of ORL emergency admissions (1). Our efforts at reduction of these preventable emergencies should be directed at heath education. Parents and caregivers need to monitor their children closely and also remove potential FBs from their vicinity.

Corrosive ingestion was a prominent indication for admission in children in this study representing 13.5% of emergency admission in children and 3.3% of the total emergency ORL admissions. This proportion was greater than 0.7 % reported in a study in Ibadan (15) Nigeria and there was no corrosive ingestion in the study done in Ghana (1) Corrosive ingestion is usually accidental in children who mistook the chemicals for water. Regulation and restrictions in the use and handling of chemical agents, their save packaging and awareness of populace about handling and storage of the chemicals (18) cannot be overemphasized as preventive strategies for reduction of corrosive ingestion.

We found that epistaxis was the major cause of emergency ORL admissions among adults similar to the report from previous published studies (7, 10). Most of the elderly patients admitted for epistaxis had associated systemic hypertension. It is an established fact that nose - bleeding is usually more severe and more difficult to control in hypertensive patients (20). The presence of arteriosclerotic plaques in the arterial walls including those of the branches of internal maxillary artery, and use of low dose aspirin medications are factors that may be responsible for this among hypertensive patients.

Vertigo was the third leading cause of adult ORL emergency admission (8.2%) among adult and 6.2% total emergency admissions. The prevalence of vertigo due to ENT cause was higher than 1.7 % reported by Lasisi *et al* (15) and much higher than 0.4% reported in India (2). The vertigos were due to peripheral vestibular causes mainly Meniere’s disease and benign paroxysmal positional vertigo. Most of them responded well to conservative management. Previous studies also reported good outcome with conservative management (2, 15).

Esophageal denture impaction was also a leading cause of ORL admissions. A similar study from Ghana shows that esophageal denture impaction represented 3.3% of ORL emergency admissions (1). Although this is less than 6.6% prevalence seen in the present study, many other studies did not report denture impaction as a cause of ORL emergencies (2, 14, 21). Denture impaction could result from carelessness in handling the dentures and failure to present for routine medical checkup even when they have noticed their denture is loose or shaking. Impaction poses a challenge to the patients and to the medical personnel due to its associated high complication. It is preventable with the proper education of the denture wearer on predisposition to, risk of impaction, denture life span, denture maintenance and the need to keep up regular visit appointment for retention assessment (24-26).

Management of ORL emergencies depends on the etiological factors. Sixty percent of our patients had one form of surgical procedure or the other. This was similar to the management protocol reported in Ghana (1). Ibekwe reported that 76 % of the elderly patients admitted for emergency ORL admission had one form of procedure or the other and that 54% of them had emergency tracheostomy. About 50% of our patients were elderly who had tumors of the larynx. One of them was in severe airway obstruction; he died at the commencement of emergency tracheostomy in the accident and emergency department. Laryngeal cancer is a highly treatable disease if presentation is early. There is a need for increased awareness of the people through social campaigns and health education on the merits of early detection and seeking appropriate treatment for these tumors (21). Among the children, upper airway obstruction was mainly due to recurrent respiratory papillomatosis. Such children presented with progressive hoarseness, stridor and respiratory distress. Early presentation and management of this condition can prevent progression into upper airway obstruction necessitating emergency admission.

Although most studies did not give detailed account of associated morbidities and complications, majority of associated complications (hoarseness, facial palsy, conductive hearing loss) found in our study resolved with time. Patients that developed esophageal strictures were referred to cardiothoracic surgery while those with facial palsy mainly grade 2 to 3 House and Beckmann stages had satisfactory outcome with physiotherapy. The mortality rate of 1.4% in our study was within the range of between 0.3 and 2.7% previously reported (1, 3, 11). The mortalities in our study were due to corrosive ingestion from deliberate self-harm, upper airway obstruction from advanced laryngeal cancer and deep neck space infections which were majorly preventable.

There are some limitations in this study which should be mentioned. The retrospective nature with its inherent problems including incomplete and loss of information is noted. Furthermore, a single hospital based study may not capture what obtains in a large country like Nigeria with diverse socio-cultural background and this is admitted a limitation. It may be necessary to extend this study to other geopolitical regions of the country.

In conclusion, the present study reveals that majority of causes for ORL admissions via emergency unit are of pharyngo-esophageal origin. There appears to be a reversal of the otological origin trend. The preventive strategies for some preventable causes of admission, and proper management protocols for most of these cases were discussed, with the hope of strengthening our health systems.
